# Supplementary material for: Are carbon emissions trading and green financial instruments synergistic? -Comprehensive quantitative research based on content analysis
Source: PLoS One. 2024 Mar 7;19(3):e0298601. doi: 10.1371/journal.pone.0298601 (PMC10919601; doi:10.1371/journal.pone.0298601)
Supplement: S1 Data — (ZIP) [file pone.0298601.s001.zip › Data/地级市Z_F工作报告.zip!/║╙─╧/╞╜╢Ñ╔╜╩╨/└┤╫╘╣1⁄2╓┌║┼í╛┬φ┐╦╩2╛▌═°í┐_2010.docx]

各位代表：

现在，我代表市人民政府，向大会报告政府工作，请予审议，并请各位政协委员提出意见。

一、2009年工作回顾

2009年，是新世纪以来我市经济社会发展最为困难的一年，也是我们砥砺奋进、逆势而上的一年。面对严峻复杂的形势，在省委、省政府和市委的正确领导下，全市上下深入贯彻落实科学发展观，坚定信心、抢抓机遇，顽强拼搏、创新克难，较好地完成了市九届人大一次会议确定的任务，保持了经济平稳较快发展，保持了走在全省前列的良好态势。

——经济运行回升向好。预计全年生产总值1140亿元，比上年增长12.1%；地方财政一般预算收入70.3亿元，增长12.5%，取得了战危机、保增长的重大胜利。

——投资消费增势强劲。城镇以上固定资产投资447.5亿元，增长28%；社会消费品零售总额294.5亿元，增长19.8%，增速均居全省前列。

——居民收入继续提高。城镇居民人均可支配收入14800元，增长9.4%；农民人均纯收入4840元，增长9.5%；城乡居民储蓄存款余额647亿元，增长21.2%，人民生活进一步改善。

——社会事业全面进步。教育、文化、卫生等事业蓬勃发展。就业、就学、就医和社会保障、住房保障实现“扩面提标”。人口自然增长率控制在5.5‰以内。

一年来，在困难和挑战面前，我们坚定目标不动摇，扭住发展不懈怠，付出了巨大努力，做了大量工作。

(一)经济运行调控积极有效。始终把保持经济平稳较快发展、保持走在全省前列的良好态势作为经济工作的首要任务，科学分析判断形势，准确把握不同阶段经济运行的突出矛盾和问题，先后六次召开经济运行会议进行动员部署，强化工作指导。紧紧抓住关键环节，创造性地开展工作，分期分批集中开工重点项目，深入开展企业服务年、大招商、产销衔接、银企洽谈等活动，极大地提振了全市上下战危机的信心。市政府与5家省级金融机构签订了1000亿元的融资协议，筹措2.3亿元中小企业担保资本金和发展资金，用于扶持企业发展和提供公共服务。年末金融机构各项贷款余额558亿元，比年初增加115亿元。市、县两级各抓100家重点企业，积极为企业排忧解难，59家停产半停产企业先后恢复生产。大力实施家电、汽车摩托车下乡等扩大消费政策，家电销售量占农户比例由年初全省第15位跃升至第2位。深入开展土地挖潜，千方百计保障重点项目建设用地。加强煤电油运调度，保障了生产生活需要。一系列应对危机的举措，有效遏制了经济下滑势头，生产总值增速从一季度的8%回升到全年的12.1%。

(二)重点项目建设成效显著。始终把项目建设作为扩内需、保增长的重要抓手，积极争取项目，强力实施“8185”投资促进工程，建立健全联审联批、市级领导联系重点项目等机制，开展观摩点评活动，逐月通报进展情况，掀起了项目建设新热潮。争取中央投资项目386个、资金10.7亿元，开工率达到100%。八大领域150个重点项目完成投资144.5亿元，为年度目标的111.2%；带动全社会固定资产投资543亿元，增长28.9%。中平能化集团30万吨离子膜烧碱及30万吨PVC树脂、市行政审批服务中心等31个项目建成投产(投用)，完成投资34.5亿元；海星化工30万吨煤焦油、叶舞高速等33个续建项目进度加快，完成投资65.4亿元；500千伏平南输变电、沙河大渡槽等48个项目开工建设，投资规模184.9亿元，完成年度投资44.6亿元；郑渝(郑州—重庆)高速铁路客运专线平顶山段、孟平铁路复线等重大基础设施项目前期工作取得积极进展。

(三)转型升级步伐明显加快。始终把调结构、促转型作为战略举措，着力构建“一个载体、三个体系”。产业集聚区建设扎实推进。10个省级产业集聚区总体发展规划编制完成，相继成立了投融资公司，健全了管理机构，完成投资69亿元，建成标准化厂房10万平方米，新开工项目55个，入驻企业500余家，载体功能初步显现。现代产业体系建设取得新进展。能源化工、装备制造、特宽厚钢、新型建材、农产品加工和现代服务业调整振兴规划正加紧编制，实施重大工业结构调整项目37个，优势产业规模和技术水平明显提升。国家电网公司与平高集团签订了重组协议，三年内投入扩建资金50亿元。中平能化集团深度融合后，重组效应凸显，营业收入突破800亿元，跨入中国企业百强行列。举办了第二届中国戏剧梅花表演奖大赛、首届中国曲剧艺术节、第八届河洛文化国际研讨会、第三届中国书法兰亭奖大赛等一系列重大活动，促进了文化产业加快发展。在全国率先推出“五一”假期景区免门票举措，激活了旅游市场，形成游客“井喷”现象。积极与中信集团、港中旅集团等央企洽谈合作，引进旅游项目19个，总投资128.1亿元。全年接待国内外游客增长46%，旅游总收入增长49%；文化旅游产业增加值占第三产业比重提高0.3个百分点。自主创新体系建设又有新突破。编制完成自主创新体系发展规划，实施了一批科技攻关项目，获国家科技进步二等奖1项，省级科技进步奖12项；申报省级重大科技专项2项、省级工程技术研究中心9家；申请专利566项，实现了国际专利申请零的突破；平高集团被认定为国家级创新型企业。高新技术产业实现增加值128亿元，增长20.8%，我市被科技部命名为科技进步先进市。节能减排力度不断加大。启动“绿色照明”工程，完成9个节能重点工程和5个工业污水治理项目。加快林业生态建设，完成造林38.6万亩，叶县和卫东区创建为省级林业生态县。万元生产总值能耗下降4.8%，化学需氧量、二氧化硫排放量分别削减2.4%和8.3%，出境水质达标率99.3%，城区大气环境质量有新的提高。

(四)农业现代化建设扎实推进。始终把“三农”工作作为基础工程，更多地关注农村、关心农民、支持农业，财政用于“三农”的投入达到12.9亿元，比上年增加4.1亿元。农业综合生产能力稳步提高。治理水土流失面积82.6平方公里，新增旱涝保收田9.2万亩、农机总动力11.8万千瓦，战胜了特大旱情等自然灾害，粮食生产连续六年获得丰收，总产达到39.1亿斤。19座中小型水库除险加固工程基本完工，北汝河治理汝州示范段开工建设。农业产业化进程步伐加快。大力发展特色高效农业、标准化规模养殖和畜产品精深加工，双汇集团25万头商品猪生产基地、伊利集团万头奶源基地开工建设，新建标准化养殖园区293个、规模养殖场233个，新申报省级农业产业化龙头企业10家，确定市级龙头企业27家，新建农民专业合作经济组织53家。新农村建设取得新进展。36个中心乡(镇)、539个中心村“两个规划”的编制评审基本完成，92个重点示范村建设全面展开，1亿元新农村建设财政补贴资金落实到位，累计投入中心村建设资金5亿元。新解决19万农民的饮水安全问题，新建农村户用沼气池3.6万座、农村公路1071公里、农家店331个。加大对农民的培训力度，完成农民工职业技能培训6.3万人。解决和巩固温饱贫困人口6.1万人，完成98个村的整村推进任务。丹江口库区第一批移民接收安置任务圆满完成，我市的做法成为全省典范。县域综合实力进一步增强。6县(市)生产总值、地方财政一般预算收入占全市的比重分别提高0.3个和0.1个百分点。

(五)城镇化水平不断提升。始终把城市创建作为改善人居环境、加快城镇化进程的重要载体，创建国家园林城市已经验收，创建国家卫生城市工作扎实推进。全市城镇化率达到41.8%，提高1.6个百分点。城乡规划加快编制。平顶山科学发展示范区(中心城区至宝丰、叶县、鲁山三个县城之间的区域)空间发展战略规划基本完善，土地利用总体规划修编已经完成，中心城区50平方公里控制性详细规划、市域村镇体系规划正抓紧编制。城镇建设强力推进。多方筹措资金，加快市政基础设施建设，市区光明路北延、迎宾路改造等12个项目竣工投用，凌云路湛河桥等7个项目正抓紧施工，中信生态文化体育公园前期工作基本完成。51个城中村和旧城开发改造项目，完成拆迁164.1万平方米、开工482.7万平方米、主体完工195万平方米，均比上年增加3倍以上，力度前所未有。房地产开发投资39亿元，增长96%，增速居全省前列。新城区建设步伐加快，大香山路、长安大道等工程正加紧施工，3万吨污水处理厂主体完工。加强湿地保护，建成国家城市湿地公园2处。县城和小城镇建设不断加强，各县(市)建成了一批市政基础设施项目，汝州市创建为中国优秀旅游城市，宝丰县通过国家卫生县城复验。城市管理日趋规范。城管体制改革成效明显，两级政府、四级管理、以区为主的城市管理体制落实到位，城乡面貌有新的改观。

(六)改革开放继续深化。始终把深化改革、扩大开放作为推动经济社会发展的强大动力，抓住关键，勇于突破，发展活力不断增强。各项改革稳步推进。市建安总公司等5家企业实现了整体转让，11家企业进入改制破产程序。农村土地承包经营权流转和集体林权制度改革步伐加快，新增土地流转面积14.2万亩，完成林地确权280.7万亩。金融体系进一步完善，成立了首家村镇银行和10家投融资公司。收入分配制度改革取得新进展，义务教育学校绩效工资开始实施。稳步推进医药卫生体制改革，基本药物制度试点工作扎实开展。大招商活动成果丰硕。成功举办了’2009华侨华人中原经济合作论坛、鹰城文化宝岛行、浙商鹰城行、第三届世界叶氏联谊会等重大活动，组团参加了国家、省举办的一系列大型经贸活动。全年签约项目176个，投资总额905亿元，其中合同资金681亿元；直接利用外资1.4亿美元，增长12.3%。鹰城文化宝岛行后，又有10多批、700多人次的台商来我市洽谈考察，台商投资园区挂牌成立。加强国际交流合作，与墨西哥圣路易斯市缔结为友好城市。平顶山已成为境内外客商竞相涌入、纷至沓来的热土，我市大招商的做法受到省政府表扬。非公有制经济进一步壮大。落实各项优惠政策，全面清理涉企收费，促进非公有制经济加快发展。非公有制经济完成增加值604亿元，增长13.5%，占生产总值比重53%，提高1.5个百分点。

(七)和谐社会建设取得新成效。始终把保障和改善民生作为发展的最终目的，向人民群众承诺的“十大实事”全部落实。就业和社会保障工作进一步加强。实施就业攻坚行动计划，城镇新增就业12.3万人，下岗失业人员再就业4.3万人，零就业家庭保持动态为零。城乡居民最低生活保障制度全面落实，9.1万人享受城市低保，17.3万人享受农村低保，人均月补差高于省定标准。新型农村合作医疗参合标准提高到100元，参合率达到94.3%。在全省率先实施敬老院建设“十个一”工程，五保对象集中供养率达到42%。鲁山县被确定为国家新型农村养老保险试点。强化住房保障，开工建设廉租房14.4万平方米、经济适用房56.9万平方米，发放廉租房租赁补贴1730万元、住房公积金贷款3.3亿元，棚户区改造基础设施配套工程完成投资1.9亿元。社会事业加快发展。落实“两免一补”资金3.2亿元，惠及学生47万人。大力度整合职业教育资源，筹资3.3亿元，以平顶山学院、河南城建学院老校区为依托，新组建可容纳万人以上的职业技术学校2所，市属职业学校由16所整合为9所，被评为全省职业教育攻坚工作先进市。积极实施文化惠民工程，建成3个县级文化信息资源共享工程支中心、改造916个村级站点，新增13个乡(镇)综合文化站和230个农家书屋。加强公共卫生设施建设，市中医院综合门诊楼竣工投用，新建改造了9个社区卫生服务中心、35个乡(镇)卫生院和500个标准化村卫生室。甲型H1N1流感和手足口病防控取得阶段性成效。继续实施“曙光行动”，率先通过“全国白内障无障碍市”省级验收。全民健身活动蓬勃开展，156个行政村体育健身工程建成使用。在第十一届全运会和第五届省残运会上，我市均取得历史最好成绩。统筹解决人口问题，出生人口性别比偏高趋势得到遏制，我市被确定为国家人口和计生综合改革示范市。加大公用事业投入，投资2700万元，更新公交车辆86台；投资3.4亿元，开工建设西气东输地方支线，实施人工煤气置换工程。社会大局保持稳定。出台了加强地方煤矿安全生产管理的十项措施和推进煤矿安全生产长效机制建设的意见，创新监管措施，强化监管责任，安全保障水平得到提高。积极开展食品药品安全专项整治，成功创建省级食品安全优秀城市。加强价格监管，物价工作综合评比全省第一。深入开展“命案侦破攻坚战”和打黑除恶、打击“两抢一盗”等专项行动，加强社会治安综合治理，治安形势明显好转，荣获第六届全国见义勇为城市奖。积极开展人民调解和法律援助，认真做好人民群众来信来访工作，解决了一批信访积案和群众关注的热点难点问题，被评为全省信访工作显著进步市。

(八)精神文明和民主法制建设得到加强。始终把加强精神文明和民主法制建设作为推动发展的重要保障，深入开展学习实践科学发展观、弘扬焦裕禄精神等活动，全市上下的向心力和凝聚力不断增强。坚持依法行政，自觉接受人大及其常委会的监督，支持政协参政议政，认真办理人大代表建议和政协提案。扎实推进“两转两提”，大力倡导“马上就办”的作风，全面推行联合审批、联合年检等“立体作业”机制，市级480个行政审批项目办理时效全部进入全省先进行列。举办政府系统秘书提高班、城市规划建设管理干部培训班、政务导游培训班，提高了公务员素质。在全省经济发展环境调查中，我市民众满意度居第2位。深入推进惩治和预防腐败体系建设，加大审计监督力度，治理商业贿赂，反腐倡廉建设取得新成效。全面推行农村“四议两公开”工作法，基层民主进一步扩大。普法和依法治理工作更加深入，全社会法治化管理水平不断提高。广泛开展群众性精神文明创建活动，城乡文明程度明显提升。

与此同时，国防教育、双拥共建、人民防空工作深入开展，军政军民关系更加巩固，荣获省级双拥模范城“四连冠”。民族宗教工作扎实推进，被国务院授予全国民族团结进步模范集体。统计工作得到加强，服务水平进一步提升。外事、侨务、对台、史志、气象、烟草、盐业、新闻出版、国家安全等工作都取得了新成效。

各位代表!

这些成绩的取得极其不易，这是省委、省政府和市委正确领导的结果，是市人大、市政协监督支持的结果，凝聚着全市广大干部群众和社会各界的心血和汗水，充分展示了鹰城儿女不畏艰难、一往无前，能够办大事、办成事的决心、勇气和智慧。实践证明，无论再大的困难，只要我们勇于面对、敢于担当，万众一心、和衷共济，就一定能够排除万难，夺取胜利!

成绩来自奋斗，功劳属于人民。在此，我代表市人民政府，向工作在全市各条战线的广大工人、农民、知识分子、干部、驻平部队指战员、武警官兵和公安民警致以崇高的敬意!向关心、支持我市现代化建设的各界人士和国际友人、港澳台同胞表示衷心的感谢!

在肯定成绩的同时，我们也清醒地看到，政府工作与人民的期望还有差距，经济社会发展中还存在一些不容忽视的矛盾和问题：一是经济回升的基础仍不稳固，工业效益和外贸出口处于较低水平。二是农业基础依然薄弱，农民持续增收难度加大，农民收入偏低。三是结构调整任务还很艰巨，能源原材料工业比重较大，第三产业比重低于全省平均水平，高新技术产业仍较薄弱。四是一些地方和部门服务意识、发展意识不强，超前谋划运作不够，项目储备不足，前期工作和基础工作不扎实。五是一些地方和企业落实“两个主体责任”不严格，隐患排查治理不到位，安全生产形势依然严峻。对此，我们一定高度重视，采取有效措施，认真加以解决。

二、2010年政府工作总体要求和目标任务

今年是“十一五”规划的最后一年，也是夺取应对国际金融危机冲击全面胜利、为“十二五”规划启动实施奠定良好基础的关键之年。经济社会发展既蕴含诸多有利条件和重要机遇，也面临较多困难和严峻挑战，但总体上看，经济发展环境将好于去年。中央继续实施积极的财政政策和适度宽松的货币政策，在保持经济平稳较快发展的基础上，重点促进发展方式转变，有利于我市加快结构优化和转型升级步伐，实现科学发展。目前我市仍处于工业化、城镇化加快推进和消费结构加快升级的阶段，总量扩张和产业升级的内在动力强劲。同时，我市产业相对完备，资源、区位等优势比较突出，随着华侨华人中原经济合作论坛等一系列重大活动的举办，我市的知名度和竞争力大大提高，有利于我们吸引更多的外商投资，借力实现跨越式发展。只要我们继续保持奋发有为的精神状态和求真务实的工作作风，创造性地开展工作，就一定能够夺取经济社会发展的新胜利!

今年政府工作的总体要求是：全面贯彻党的十七大和十七届三中、四中全会精神，以邓小平理论和“三个代表”重要思想为指导，深入贯彻落实科学发展观，认真落实中央、省委和市委经济工作会议各项部署，继续推进“生态建市、产业立市、文化强市、和谐兴市”战略，学先进、比创新、看实效，把加快经济发展方式转变作为重要目标和战略举措，大力实施“新农村建设、城镇化发展、现代服务业发展”三大提速工程，着力优化产业结构，着力统筹城乡发展，着力深化改革开放，着力保障改善民生，着力促进社会和谐，全面完成“十一五”目标，精心谋划“十二五”发展，持续保持经济平稳较快发展、持续保持走在全省前列的良好态势。

经济社会发展的主要预期目标是：生产总值增长12%；地方财政一般预算收入增长13%；全社会固定资产投资增长21%；社会消费品零售总额增长15%；外贸出口增长11%；实际利用外资增长15%；居民消费价格涨幅控制在3%左右；城镇居民人均可支配收入实际增长8%，农民人均纯收入实际增长6%；人口自然增长率控制在6.5‰以内；城镇新增就业6.2万人，城镇登记失业率控制在4.5%以内；单位生产总值能耗降低4.4%，化学需氧量、二氧化硫排放量分别下降1.5%和2.6%；第三产业比重和城镇化率均提高2个百分点以上。

扎实做好“十大民生工程”。筹措资金30亿元，重点解决事关人民群众切身利益的就业、就学、就医、社会保障、住房保障、农民增收等方面的问题，让经济社会发展的成果由人民共享!

实现上述目标，我们必须坚持“重在持续、重在提升、重在统筹、重在为民”的要求，切实做好八项工作：

(一)落实完善扩大需求政策措施，促进经济平稳较快发展。继续抓住需求不足这一主要矛盾，完善调控措施，扩大需求规模，进一步巩固经济回升向好的态势。

强力推进重点项目建设。在能源化工、装备制造、节能减排、社会事业等8大领域，安排实施170个投资千万元以上的重点项目，总投资规模1500亿元，年度投资150亿元，带动全社会固定资产投资600亿元以上。建成鲁阳电厂一期2×100万千瓦机组、500千伏平南输变电、叶舞高速等59个项目，完成投资80亿元；续建新平高特高压超高压开关、南水北调中线工程平顶山段、北汝河综合治理等32个项目，完成投资20亿元；开工建设舞钢公司热铁直送技术改造、中平能化集团夏店矿、汝登高速平顶山段等55个项目，完成投资50亿元；积极做好平顶山至许昌城际轨道交通、尧山机场、尧西(尧山—西峡)高速等24个项目前期工作，争取开工郑渝高速铁路客运专线平顶山段。继续坚持市级领导联系重点项目等协调推进机制，优化建设环境，及时解决问题，确保早开工、早投产、早见效。加强对财政投入项目的跟进审计、全程监督，防止出现“半拉子”工程和豆腐渣工程，从源头杜绝腐败现象。

积极扩大消费需求。更加注重扩大消费与改善民生相结合，进一步增强消费对经济增长的拉动作用。完善企业工资集体协商和工资支付保障制度，提高企业退休人员基本养老金、部分优抚对象待遇、城乡居民最低生活保障水平，落实义务教育学校、医疗卫生等公益性事业单位绩效工资政策，努力增加居民收入。加快专业市场、特色商业街和多功能商贸服务设施建设，积极发展电子商务、专业配送等新型商贸流通业态，大力推动健身、家政、养老等服务消费。继续实施“万村千乡市场工程”和“新网工程”，落实家电、汽车下乡等优惠政策，积极发展消费信贷，促进消费增长。整顿和规范市场秩序，深入开展食品药品安全专项整治，维护消费者合法权益。

切实加强经济运行调节。把握好调控的节奏和重点，推动经济平稳健康运行。深化企业服务活动。建立健全政策导向、市场开拓、融资服务、困难企业帮扶等长效机制，推动企业服务工作常态化。支持、引导外地企业来平登记注册，设立分支机构，把新登记注册企业数量纳入政府目标考核体系，加快培育市场主体。市、县两级继续各抓100家重点企业，建立健全企业服务中心，把服务企业与行风政风评议结合起来，切实提升服务效率和水平，努力在全社会营造“亲商、重商、富商”的浓厚氛围。强化财税金融支持。落实积极的财政政策，扩大政府投资，引导带动社会投资；优化财政支出结构，加大对“三农”、民生、自主创新、节能环保、技术改造等方面的支持力度，控制一般性支出，确保有限的资金用在最需要的地方。落实各项税费优惠政策，减轻企业和居民负担；依法加强税收征管和非税收入管理，严厉打击逃避纳税行为。用好适度宽松的货币政策，提高市县政府融资能力，扩大直接融资规模，缓解中小企业融资难问题。加强经济运行监测和要素供需协调。密切关注国家宏观调控政策和市场变化，及时研究实施针对性措施。加强煤电油运供需调度，确保正常生产生活需要。建立健全应急事件处置预案和协调服务机制，提升应急保障能力，最大限度地减少因要素供给不力而影响企业生产经营。加强市场监管，保持物价基本稳定。

(二)大力推进经济结构战略性调整，提高经济增长质量和效益。围绕构建现代产业体系，编制实施能源化工、装备制造等六大战略产业发展规划，加快转型升级项目建设，促进产业结构优化升级，提升产业竞争力。

加快产业集聚区建设。按照创建品牌集聚区的要求，出台《产业集聚区管理办法》，制订异地投资税收分成办法，建立项目进度定期通报制度，进一步规范产业集聚区发展。多渠道筹集资金，加快公共服务设施建设，提升产业集聚区的吸纳承载能力。以主导产业为依托，拉长产业链条，大力发展优势产业集群。全年10家省级产业集聚区完成投资100亿元，新开工标准化厂房40万平方米，新入驻千万元以上企业50家，实现销售收入800亿元以上。启动10个市级产业集聚区的规划建设，健全推进机制，培育特色主导产业，带动相关产业发展。

推进工业结构优化升级。改造提升传统产业。以骨干企业为依托，加快技术改造步伐，强化技术创新的支撑作用，提高装备水平和生产能力，再造竞争新优势。支持中平能化集团挺进世界500强。建成20万吨尼龙66盐成套设备、洁石建材年产2800万平方米高档陶瓷砖等23个项目；续建1万吨锦纶66中低旦工业丝等11个项目；开工建设60万吨尼龙6一期、平煤机高端液压支架等11个项目；加快叶县10万千瓦风能发电等项目前期工作。培育发展战略性新兴产业。以新能源、新材料、电子信息和生物医药等新兴产业为重点，加快产业链前端产品研发和后端产品推广应用，抢占新一轮发展先机。新增市级以上高新技术企业10家，高新技术产业增加值增长15%以上。加快推进信息化和工业化深度融合，选择重点行业和龙头企业开展“两化融合”试点。加快淘汰落后产能。健全部门协调联动机制，全部拆除已关停的小砖瓦窑、小石灰窑、小炼铁等落后生产设施，全部关闭不具备安全条件的小煤矿，改造关闭不符合准入标准的新型墙材生产线。按照全市焦化行业整合意见，建成7条捣固焦生产线，淘汰落后产能。

大力发展现代服务业。以文化旅游产业为龙头，以100个投资千万元以上、10个投资亿元以上的服务业发展项目为抓手，实施“现代服务业发展提速工程”，提高第三产业在生产总值中的比重。编制实施尧山旅游休闲度假区、汝州文化旅游特色园区发展规划，深化与中信集团、港中旅集团等中央和国内外知名企业的合作，加快以旅游交通、游客服务、旅游安全为重点的基础设施建设，努力把尧山旅游休闲度假区打造成国际精品旅游景区，把我市打造成国内重要、国际知名的旅游目的地。实施大香山风景名胜区发展规划，扩建三苏园，建设中原盐湖度假村等文化旅游项目。加大宣传推介力度，积极申办全国休闲旅游节、摄影大赛和诗歌大赛等活动，唱响“山水鹰城、近悦远来”主题口号，进一步提升旅游整体形象和市场占有率。加快现代物流业发展，编制完成三大综合物流园区发展规划，建成平东煤炭物流中心等项目，健全农产品进城、工业品下乡流通网络。推动组建农村商业银行，积极发展村镇银行，鼓励支持市商业银行跨区域经营，引进股份制商业银行及证券、保险公司在我市设立分支机构，加快金融保险业发展。吸引有实力的房地产商参与我市城中村开发改造，大力推进物业管理进社区，促进房地产业平稳健康发展。积极推动社区服务、商务中介、信息咨询等新兴服务业发展。

(三)加快转变发展方式，增强综合竞争力和可持续发展能力。坚持在发展中促转变，在转变中谋发展，务求在一些重要领域和关键环节实现新突破。

切实提升自主创新能力。实施质量兴市战略，加快培育创新型企业，筹建国家级高压电气设备质检中心，覆盖中南地区乃至国际市场高压电气产品的研发检测。加强企业研发中心建设，新增省级工程技术研究中心3家、省级以上重点实验室1个、省级企业技术中心5家。强化知识产权保护，进一步提高专利申请数量和质量。大力实施名牌战略和标准化战略，争创国家和省名牌产品3个、省优质产品5个以上，完成产品采标确认3个、计量合格确认10家，不断提升市场竞争力和品牌影响力。依托科研机构、重大科研和工程项目，培养、引进技术创新骨干人才，建设高层次科技人才队伍。

大力推进节能减排。完善节能减排考核体系，严格实行“一票否决”制度。强化重点领域节能。在工业领域实施“节能行动计划”，通过强化管理、加大投入、挖潜改造，努力实现全年节能65万吨标准煤目标。建立投资项目节能评估制度，从源头控制高耗能、高排放项目发展。坚持最严格的耕地保护和节约用地制度，严禁违法用地，确保重点建设用地。加快建设节水工程，推进矿井水、地热等资源化利用。严格落实建筑节能标准，大力推广新型墙材。推进公共机构节能减排，建设节约型机关。积极发展循环经济。在企业、园区、城镇三个层面发展循环经济，争取列入省级循环经济试点市、2家产业集聚区列入省级试点。实施一批废弃物利用、再生资源回收项目，力争工业固体废弃物综合利用率达到75%以上。加强生态建设和环境保护。扎实推进“生态鹰城”建设，新增造林19.8万亩，完成3个省级林业生态县、20个林业生态乡、300个林业生态村的绿化创建任务。积极发展低碳经济，努力控制温室气体排放。巩固燃煤锅炉整治成果，推广使用天然气等清洁燃料，不断提高市区空气质量。加强饮用水源地保护，高度重视南水北调工程沿线的环保，确保水质达标，让人民群众喝上安全水。完善在线监控设施，污水处理厂运行负荷率达到80%以上。

努力实现安全发展。牢固树立安全发展理念，启动“安全鹰城”创建。加大安全教育培训力度，提高全民安全素质。切实把煤矿安全生产放在突出位置，从源头治理、强化责任、加强管理、综合防控等方面入手，多策并举，推动地方煤矿兼并重组，加快整合步伐，提高产业集中度和安全保障水平。深入开展非煤矿山、道路交通等重点领域和商场、学校等人员密集场所隐患排查治理，建立安全投入保障机制，坚决遏制重特大事故发生。

(四)扎实推进新农村建设，提升农业现代化水平。坚持把解决“三农”问题作为重中之重，实施“新农村建设提速工程”。着力提高粮食综合生产能力。继续实施“粮食生产五年增长计划”，深入开展“百千万”高产创建示范活动和千名科技人员包千村活动，粮食种植面积保持在610万亩以上，总产增长5%左右。落实种粮直补和主要粮食品种最低收购价政策，保护和调动农民种粮积极性。进一步提升农业产业化水平。大力发展现代畜牧业，突出抓好标准化、规模化养殖，新建各类标准化养殖园区150个。加强动物疫病防控体系建设，提高质量安全水平。积极引进大型农产品加工企业，新增农产品加工项目10个。做大做强龙头企业，新增省级龙头企业3家、市级龙头企业10家，新增农民专业合作经济组织50家。努力改善农村生产生活条件。全面落实农机补贴政策，提高机械化耕作水平。抓好中低产田改造和农田水利建设，加强气象灾害监测预警，提高抗灾减灾能力。切实做好农业科技信息服务工作，建成市级农业科技信息服务中心。增加并整合支农资金，加强农村基础设施建设，新建改建农村公路380公里，新增大型沼气工程15个、沼气用户2万户，新解决农村饮水安全18万人，新建农家店300家以上。加大对贫困地区特别是革命老区的扶持力度，解决和巩固温饱贫困人口6万人，完成80个村的整村推进任务。加大农村劳动力转移就业培训力度，新增转移就业6.5万人。积极推进中心村建设。全面落实“两个规划”，筹措资金1亿元，继续扶持中心村和农民住房建设，使92个示范村面貌发生根本性改变。建立科学推进机制，重视发挥示范户的带动作用，落实中心村建房贷款贴息等政策，加快新农村建设进程。

(五)着力统筹城乡发展，加快城镇化进程。实施“城镇化发展提速工程”，坚持以产业为基础，以产业化带动城镇化，通过产业集聚区发展，提升城镇化水平。发挥产业集聚区带动作用。以产业集聚区建设为载体，吸引人才、资金、技术等生产要素向县城和重点镇集聚，形成集聚促进就业、就业创造消费、消费拉动经济增长的良性发展，逐步把县城发展成为20万人以上的中等城市。积极推进户籍制度改革，研究政策，放宽城镇落户条件。加快科学发展示范区建设。高标准编制规划，上半年完成编制和报批。示范区内，按城市社区规划建设。建成长安大道，推进平叶、平鲁快速通道建设，规划建设平郏快速通道，加快沙河两岸景观设计，形成以市区为中心、直达县城的20分钟交通圈。支持舞钢市城乡一体化试点工作。加大城市基础设施建设力度。筹措资金40亿元，实施20项市政基础工程，规划建设大南环、大北环路，完善路网结构，拉大城市框架。加快城中村和旧城开发改造步伐，完成1850万平方米改造任务，积极推进平安大道北部3.5平方公里区域旧城改造。在人员密集、出行不便的地方，规划建设过街天桥。巩固创建国家园林城市成果，提高城市绿化水平。大力开展爱国卫生运动，清洁城乡，保护健康，力争创建为国家卫生城市。投资20亿元用于新城区建设，着力完善医院、商业网点等配套服务设施，提升承载能力，加快聚集人气。积极发展公共交通，改善人民群众出行条件。实施城市精细化管理。巩固城管体制改革成果，实现城管重心下移。修订完善作业标准，建立考核评价体系，推进数字城管建设。加强宣传教育，引导市民增强城市意识，自觉遵守交通规则。实施绿、亮、净、美、畅工程，推动城市管理精细化、标准化。提升县域经济发展水平。进一步落实和完善支持县域经济发展的各项政策，加大扶持力度，引导各县(市)大力发展特色经济、民营经济和劳务经济，优化产业结构，壮大经济规模，努力在全省县域综合实力排名中位次前移。

(六)继续深化改革开放，破解经济社会发展难题。着力解决制约科学发展的突出矛盾和问题，坚持改革创新，提高对外开放水平，增强经济社会发展动力和活力。深入推进各项改革。继续深化国有企业改革，力争完成酿酒总厂、五金矿产公司等已进入程序的企业改制任务，强力推进商贸、物资等流通企业改革。完善县域土地流转有形市场，新增流转面积10万亩以上。加快林地确权步伐，完成集体林权制度改革。实施上市后备企业培育工程，争取天瑞水泥、中加公司、易成新材料公司实现上市。加快医药卫生体制改革，完善基本医疗保障制度，全面实现医疗救助与新农合、城镇职工和居民医保同步结算。积极推动企业战略合作。以平高集团与国家电网公司战略重组为契机，推动输配电装备制造产业化，打造中原电气城。支持东方希望集团发展盐化工，支持煤电、建材、钢铁、煤盐化工等企业进行战略合作和重组。继续开展大招商活动。组团参加国家、省重大招商活动，有针对性地到省外、境外进行招商，积极申办’2010华侨华人中原经济合作论坛，吸引和承接国内外高层次产业转移。进一步加强对签约项目的跟踪服务，推动一批重大合作项目尽快落地。努力扩大出口规模。加大进出口经营权登记申报力度，支持更多企业走向国际市场。加强对中平能化集团、舞钢公司等外贸骨干企业的跟踪扶持，力争再培育2个出口超千万美元的拳头产品。优化出口产品结构，深度开发传统市场，努力开拓新兴市场，培育新的出口增长点。大力发展非公有制经济。认真落实优惠政策，充分发挥中小企业发展专项资金引导作用，着力扶持100户重点非公企业做大做强。

(七)切实加强文化建设，加快发展文化产业。充分发挥文化资源优势，搞好整体策划，提升发展水平，建设文化强市。大力繁荣文化事业。围绕我市特色文化，创作高水平的舞台剧，打造舞台艺术精品。强力推进县(市)图书馆、乡(镇)综合文化站、文化信息资源共享、广播电视村村通、农家书屋等文化惠民工程，继续开展舞台艺术送农民和农村电影公益放映等活动。加快推进博物馆和文化艺术中心配套设施建设。着手筹建新的市广播电视发射塔和大型多功能体育馆，年内完成选址和规划设计。高等院校图书馆对社会开放。规划建设新的市老干部活动中心和老干部大学。大力弘扬书法艺术，推动各类学校开设书法选修课，创建“中国书法城”。加强重点文物和非物质文化遗产保护及开发利用。广泛开展全民健身活动，积极备战第十一届省运会。加快发展文化产业。集中实施一批重大文化产业项目，加快推进中信生态文化体育公园、昊澜国际生态产业园、汝瓷文化产业园建设。实施宝丰县文化改革发展试验区规划，加快建设马街书会风情园、赵庄魔术大观园，打造在全国有较大影响的魔术演艺产品交易中心。积极开发地方特色文化旅游商品，拓展文化市场，壮大产业规模。举办第五届中国宝丰魔术文化节和首届中国汝瓷文化节。继续深化文化体制改革。积极推进公益性文化事业单位改革，加快文化市场综合执法改革，完成市豫剧团、曲剧团、越调剧团改制。拓宽文化产业融资渠道，鼓励民间资本进入文艺表演、文化娱乐、出版物分销等领域。切实加强精神文明建设。继续深入开展学习实践科学发展观活动。大力弘扬爱国主义、集体主义、社会主义思想，加强社会公德、职业道德、家庭美德和个人品德教育。广泛开展创建文明单位、文明社区、文明景区竞赛活动，继续推进科教、文体、法律、卫生进社区和文化、科技、卫生“三下乡”活动，提高城乡文明程度。

(八)积极推进民生工程建设，着力构建和谐社会。改善民生是最大的政治，我们要下大力气解决关系人民群众切身利益的问题。努力扩大社会就业。完善促进全民创业的政策和服务体系，继续发挥政府投资和重大项目带动就业的作用，提高中小企业、劳动密集型产业和服务业吸纳就业的能力。进一步开发公益性岗位，重点帮扶城镇“4050”群体、高校毕业生和农民工就业，力争下岗失业人员再就业2.5万人，动态消除“零就业家庭”。不断提高社会保障和住房保障水平。扩大社会保险覆盖面，推动城镇职工基本医疗保险、失业保险、工伤保险市级统筹，基本解决各类关闭破产企业退休人员和困难企业职工医疗保障问题。加强社保基金管理，确保基金安全。在全省率先建立低收入群体临时救助制度，对基本生活出现暂时困难的低保边缘家庭给予临时救助。着力改善残疾人生产生活条件，争创全国残疾人工作示范市。开展多种形式的慈善活动，救助更多的困难群众。加快保障性住房建设，建成经济适用房30万平方米、廉租房10万平方米，新开工经济适用房79万平方米、廉租房19.1万平方米。完成棚户区改造配套设施建设，加快城市和国有工矿棚户区改造。大力发展教育、卫生等社会事业。编制全市教育事业中长期发展规划和中小学布局规划，着力促进教育均衡发展。进一步规范办学行为，逐步解决城区中小学大班额问题。加快校舍安全工程建设，完成加固和改造面积52万平方米以上。大力推进职业教育攻坚，扩大优质职业教育资源，扩大招生规模，对中等职业学校农村家庭经济困难学生和涉农专业学生免除学费，实现普及高中阶段教育目标。推进职业教育强市(县)创建工作，打造职教“示范学校”和“品牌专业”。鼓励社会办学，促进民办教育发展。支持高等院校建设和发展，努力办出特色、办出水平。完善基层医疗卫生服务体系，加强疾病控制等卫生机构基础设施建设，继续搞好甲型H1N1流感、手足口病等传染病防控。落实计划生育综合治理措施，保障计划生育家庭权益，稳定低生育水平，提高出生人口素质，巩固示范市创建成果。努力维护社会稳定。深入推进社会管理创新，正确处理各类社会矛盾，积极预防和妥善处置群体性事件和突发公共事件。认真做好民族宗教工作，确保和谐稳定。充分利用国家政策，筹措资金1.6亿元，加强政法队伍装备建设和经费保障，在市内三区组建1000人的城市巡防队伍。强化社会治安综合治理，依法严厉打击各类违法犯罪活动，不断提高人民群众的安全感和满意度。做好普法、依法治理、人民调解和法律援助工作，加强行政复议和仲裁，努力建设“法治鹰城”。完善村民自治，强化村务公开和民主管理。加强社区建设，完善服务功能，提高服务水平，创建和谐社区。建立健全信访工作长效机制，坚持月考评制度，切实加强市直各部门和基层单位的信访工作，强化责任追究，确保进入全省先进行列。

深入开展国防教育和动员，支持驻平解放军和武警部队建设，不断提高民兵预备役部队和人民防空建设质量；积极开展双拥共建活动，巩固和发展军政军民团结，争创全国双拥模范城“四连冠”。搞好第六次全国人口普查，加强统计监测服务工作。支持工会、共青团、妇联等人民团体，充分发挥桥梁纽带作用和参与社会事务管理的作用。继续做好外事、侨务、对台、史志、盐业、国家安全、防震减灾等工作，全面完成江油恢复援建任务。

三、进一步加强政府自身建设

顺应经济社会发展新形势、新要求和人民群众的新期待，以“两转两提”为重点，着力建设“学习型、创新型、法治诚信型、服务型、廉洁型”政府，切实提高运作能力和水平。

(一)坚持勤奋学习，建设学习型政府。建立健全公务员培训制度，按照“能写、能讲、能干、会做群众工作”的要求，进一步加大培训力度，着力提高公务员素质。各级政府工作人员特别是领导干部，要树立终身学习观念，把学习作为增长才干的重要途径，作为提高执行力、创造力、运作能力的重要手段，带头加强学习、勤于思考、学以致用，及时了解中央和省的决策部署，结合实际，贯彻落实，把更多的精力放在分析问题、研究问题、解决问题上，不断提高推动科学发展的能力。 (二)坚持开拓进取，建设创新型政府。应对危机的关键阶段，是对各级干部创新能力的最好检验。要进一步解放思想，培养创新意识，增强创新勇气，提高创新能力，切实把创新成果体现在加快发展方式转变上。要以政府机构改革为契机，进一步创新行政管理体制，完善工作方式和运行机制，以创新的思路和举措，推进重点工作和热点难点问题的解决，切实把创新型政府建设贯穿于促进经济社会又好又快发展的全过程。

(三)坚持依法行政，建设法治诚信型政府。严格按照法定权限和程序行使权力、履行职责，依法管理经济社会事务。完善政府决策、执行、监督机制，健全重大事项集体决策、专家咨询和公示、听证等制度。落实行政执法责任制和评议考核制，严格执行绩效管理和问责制度，加大对滥用权力和失职渎职行为的追究力度，严肃整治行政不作为和乱作为。自觉接受人大及其常委会的法律监督、工作监督和政协的民主监督，重视新闻舆论和社会监督，认真办理人大代表建议和政协提案，不断提高落实率和满意度。坚持说到做到、有诺必践，言必信、行必果，提高政府公信力。以政府信息公开为重点，进一步拓宽政务公开的渠道和范围，做到政策公开、程序规范、运作透明。

(四)坚持务实高效，建设服务型政府。牢固树立公务员就是服务员的理念，坚持求真务实、力戒空谈，扎实做事、干事成事，务求实效。积极推行标准化服务，深入开展“人民满意的公务员”评选活动，努力为基层、为群众提供优质、高效的服务。精简会议、文件，改进会风、文风。大力提倡“马上就办”的作风，进一步完善联合审批、联合年检等“立体作业”机制，最大限度地减少环节、提高效率，使全市所有审批事项的办结速度达到全省一流。建立健全应急管理体制，提高应对突发公共事件的能力和水平。

(五)坚持执政为民，建设廉洁型政府。着力推进反腐倡廉制度建设，教育引导各级干部带头学习制度、严格执行制度、自觉维护制度，不断提高制度执行力。认真落实党风廉政建设责任制，加强监察、审计等专项监督，做到关口前移，扎实推进惩治和预防腐败体系建设。各级干部特别是领导干部，要常怀对公共权力的敬畏之心，严格要求自己，加强党性修养，心系群众、关注民生，勤俭节约、勤俭办事，努力在全社会、在老百姓心中树立公道正派、团结干事、廉洁高效、务实为民的良好形象，以优良的政风带民风，让大家在风正气顺的和谐环境中干事创业!

各位代表!

莫道昔日寒未尽，明朝春色倍还人。战危机考验了我们，战危机更坚定了我们的信心。把平顶山建设得更加美好，是500万鹰城人民的期待和重托，是时代赋予我们的责任和使命。让我们以更加昂扬的斗志、更加扎实的作风，锲而不舍、开拓创新、团结奋进，为我市持续保持经济平稳较快发展、持续保持走在全省前列的良好态势而努力奋斗!
